# Epidural and transcutaneous spinal cord stimulation facilitates descending inputs to upper-limb motoneurons in monkeys

**DOI:** 10.1088/1741-2552/abe358

**Published:** 2021-03-22

**Authors:** Thomas Guiho, Stuart N Baker, Andrew Jackson

**Affiliations:** jne1 Biosciences Institute, Faculty of Medical Sciences, Newcastle University, Newcastle NE2 4HH, United Kingdom; andrew.jackson@ncl.ac.uk

**Keywords:** spinal cord injury, non-human primates, spinal networks, spinal cord stimulation, upper-limb movements

## Abstract

*Objective.* There is renewed interest in epidural and transcutaneous spinal cord stimulation (SCS) as a therapy following spinal cord injury, both to reanimate paralyzed muscles as well as to potentiate weakened volitional control of movements. However, most work to date has focussed on lumbar SCS for restoration of locomotor function. Therefore, we examined upper-limb muscle responses and modulation of supraspinal-evoked movements by different frequencies of cervical SCS delivered to various epidural and transcutaneous sites in anaesthetized, neurologically intact monkeys. *Approach.* Epidural SCS was delivered via a novel multielectrode cuff placed around both dorsal and ventral surfaces of the cervical spinal cord, while transcutaneous SCS was delivered using a high carrier frequency through surface electrodes. *Main results.* Ventral epidural SCS elicited robust movements at lower current intensities than dorsal sites, with evoked motor unit potentials that reliably followed even high-frequency trains. By contrast, the muscle responses to dorsal SCS required higher current intensities and were attenuated throughout the train. However, dorsal epidural SCS and, to a lesser extent, transcutaneous SCS were effective at facilitating supraspinal-evoked responses, especially at intermediate stimulation frequencies. The time- and frequency-dependence of dorsal SCS effects could be explained by a simple model in which transynaptic excitation of motoneurons was gated by prior stimuli through presynaptic mechanisms. *Significance.* Our results suggest that multicontact electrodes allowing access to both dorsal and ventral epidural sites may be beneficial for combined therapeutic purposes, and that the interaction of direct, synaptic and presynaptic effects should be considered when optimising SCS-assisted rehabilitation.

## 1. Introduction

Spinal cord injuries (SCIs) have disastrous consequences for patients who face major visceral and motor impairments (Snoek *et al* 2004). Recent years have seen considerable progress in the use of spinal cord stimulation (SCS) to restore locomotor function. This was initially conceived as an alternative to direct functional electrical stimulation (FES) of muscles (Taylor, 2002, Guiraud, 2006) to engage surviving central pattern generator (CPG) circuitry deprived of supraspinal control (Dimitrijevic *et al* 1998, Minassian *et al* 2004). However, clinical trials of epidural lumbar SCS additionally revealed unexpected improvements in volitional control of the lower limbs, for example the ability to voluntarily lift the legs while lying down, even for people with injuries classified as clinically complete (Harkema *et al* 2011, Angeli *et al* 2018). This suggests that many clinically-complete lesions may nevertheless be anatomically incomplete, and that SCS can raise the excitability of spinal circuits to unmask weakened but surviving descending pathways (Minassian *et al* 2016). In conjunction with extensive rehabilitation, these pathways may be strengthened to further support restoration of function (Van Den Brand *et al* 2012, McPherson *et al* 2015, Krucoff *et al* 2016, Duffell and Donaldson 2020).

If one therapeutic mechanism of SCS is raising spinal excitability rather than directly driving CPGs, this technique could also be effective for restoration of upper-limb function, identified as a top priority by patients with quadraplegia (Anderson 2004). Control of the upper-limb in humans and non-human primates relies heavily on corticospinal neurons, many of which synapse directly onto cervical motoneurons via the pyramidal tracts (Lemon 2008). In principle, the appropriate pattern of cervical SCS could raise motoneurons closer to threshold enabling their activation by weakened descending pathways. Although previous studies in non-human primates have examined the ability of cervical SCS to drive muscles directly (Moritz *et al* 2007, Zimmermann *et al* 2011, Sharpe and Jackson 2014, Greiner *et al* 2021, Kato *et al* 2020), little is known about the interaction between SCS and descending motor commands. It is not clear whether cervical SCS protocols (electrode locations, stimulus frequency, pattern etc) optimized for transiently activating muscles directly will necessarily prove best for providing sustained elevation of spinal excitability. For example, Sharpe and Jackson (2014) demonstrated that while stimulation of the ventral surface of the spinal cord was effective at driving upper-limb muscle responses, it was less effective than dorsal surface stimulation at facilitating the response to a subsequent intraspinal stimulus. The primary aim of our study was therefore to examine the interaction between trains of epidural SCS, delivered at different frequencies to various sites around the cervical spinal cord, and supraspinal inputs, elicited by stimulating the motor cortex or pyramidal tract. To this end we used a novel epidural cuff electrode that allowed current to be delivered through eight contacts located around the circumference of the spinal cord, providing a means to stimulate both dorsal and ventral aspects.

Recently, transcutaneous SCS has emerged as a non-invasive method for therapeutic stimulation (Megía García *et al* 2020). Often this is delivered using the ‘Russian Current’ method, in which low stimulation frequencies modulate a higher carrier frequency that is thought to reduce the pain associated with high stimulation intensities (Ward 2009). While transcutaneous SCS has shown promising benefits when delivered at both lumbar (Gerasimenko *et al* 2015, Hofstoetter *et al* 2015) and cervical (Gad *et al* 2018, Inanici *et al* 2018, Benavides *et al* 2020) levels, the mechanism of action is unclear. A secondary aim of our study was therefore to examine the facilitatory effects of transcutaneous SCS on supraspinal inputs to the spinal cord and compare this with epidural SCS.

## 2. Methods

### 2.1. Surgical methods

Experiments were performed on five anesthetized, neurologically intact monkeys (monkey Si—7 years, 6.6 kg; Un—5 years, 5.9 kg, Uk—7 years, 8.8 kg, Yi—5 years, 7.5 kg and Yu—4 years, 5.3 kg) under appropriate UK Home Office licenses in accordance with the Animals (Scientific Procedures) Act 1986, and with the approval of the Animal Welfare and Ethical Review Board of Newcastle University. We report data from terminal experiments involving epidural SCS, performed under an anaesthetic regime involving intravenous infusion of ketamine (6 mg kg^−1^ h^−1^), alfentanil (0.2–0.3 *μ*g kg^−1^ min^−1^) and midazolam (0.14 mg kg^−1^ hr^−1^). We have used this regime previously (e.g. Sharpe and Jackson 2014), since ketamine does not depress spinal excitability to the same extent as volatile agents (Kendig 2002). Temperature, heart rate, saturation, blood pressure and end-tidal CO_2_ were monitored throughout and at the end of the terminal procedure animals were perfused transcardially with buffer followed by formalin fixative. In addition we report data from recovery sedation sessions involving transcutaneous SCS in Monkeys Uk and Yu. Sedation was maintained with an intravenous infusion of ketamine (6.5 mg kg^−1^ h^−1^), midazolam (0.33 mg kg^−1^ h^−1^) and medetomidine (0.001 mg kg ^−1^h^−1^) while temperature, heart rate, saturation and blood pressure were monitored.

#### 2.1.1. Epidural SCS

**Figure 1.**
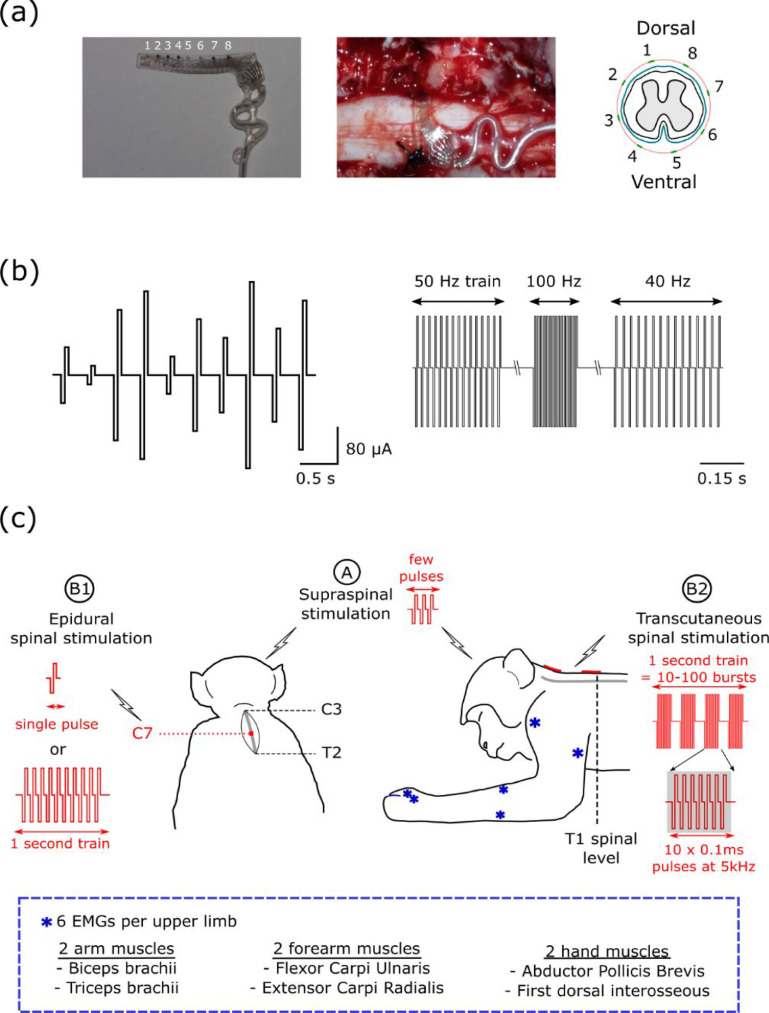
Experimental set-up. (a) Cuff electrode for epidural stimulation of dorsal and ventral cervical spinal cord (C7 spinal level). (b) Stimulus patterns used to characterize the effect of intensity (left) and frequency (right). (c) Paired stimulation protocols. Supraspinal intracortical microstimulation or pyramidal tract stimulation (A) was paired with spinal cord stimulation delivered either through epidural electrodes (B1) or transcutaneous electrodes (B2). EMG responses were recorded from six upper-limb muscles on each side.

Epidural SCS was delivered using an eight-contact electrode array placed around the circumference of the cervical spinal cord (AirRay Fetz Spinal Cord 8, Cortec Germany; 0.3 mm diameter platinum electrodes, 2.5 mm pitch on a 27 × 2.5 mm silicone substrate). Under initial sevoflurane anaesthesia, a laminectomy was made to expose the cervical enlargement. A pliable guide-rod was used to tunnel a catheter under the spinal cord at the C7 level. Sutures passed through the catheter were used to pull the spinal electrode underneath the cord and tie it snugly around the dura (figure 1(a)).

#### 2.1.2. Electromyogram recording

Pairs of Teflon-insulated stainless steel wires were inserted percutaneously to record evoked potentials from 12 muscles (bilaterally: 1DI, first dorsal interosseous; APB, abductor pollicis brevis; ECR, extensor carpi radialis; FCU, flexor carpi ulnaris; BB, biceps brachii; TB, triceps brachii). Electromyogram (EMG) signals were amplified with a gain of 1000, band pass filtered between 100 Hz and 1 kHz (Model 1700, A-M Systems, Carlsborg, US) and sampled at 5 kHz (Micro 1401, CED Cambridge, UK).

#### 2.1.3. Supraspinal stimulation

Intracortical microstimulation (ICMS) was delivered through tungsten microelectrodes (Microprobes, US) inserted through a craniotomy over the hand area of primary motor cortex, positioned to elicit low threshold responses in contralateral arm/hand muscles. In two animals (monkey Yi and Yu) we also positioned stimulating electrodes in the pyramidal tract (stereotaxic coordinates A2.0, L1.5 and P3.0, L1.5). These electrodes were fixed at a height which maximized the short-latency antidromic field potentials recorded from the motor cortex after stimulation through the electrode.

#### 2.1.4. Transcutaneous SCS

Transcutaneous SCS was delivered in two animals (monkeys Uk and Yu) during repeated sessions under light sedation. At the beginning of each session, the skin was shaved and cleaned before the cervicothoracic junction was identified by skin palpation. Two transcutaneous adhesive electrodes (circular, 2.5 cm diameter, Model J10R00, Axelgaard Manufacturing, Denmark) were positioned above C3-C4 and C7-T1 intervertebral spaces.

### 2.2. Stimulation protocols

#### 2.2.1. Intensity series

Single biphasic, constant current pulses (0.1 ms per phase, cathodal first) were delivered through the spinal epidural contacts (using a return electrode placed in nearby muscle) with an isolated, constant current stimulator (Model DS4, Digitimer, Hertfordshire, UK). Initially we used ten stimulus intensities of 20, 40, …, 200 *μ*A. For each contact on the electrode, 20 repetitions of each intensity were delivered in a pseudorandomized order with an interstimulus interval of 0.5 s (figure 1(b)). Depending on the threshold for muscle activation, additional series (5–50 *μ*A, 50–500 *μ*A, 200–2000 *μ*A) were performed as appropriate.

#### 2.2.2. Frequency series

Dorsal and ventral spinal epidural contacts were then stimulated with trains of 15 pulses at frequencies between 10 and 120 Hz (10 Hz increments) using a different isolated constant-current stimulator (Model 2100, A-M Systems, Carlsborg, US). Twenty repetitions of each frequency were delivered in a pseudorandomized order, with a one second interval between each train (figure 1(b)). The stimulus intensity was set slightly above threshold so as to elicit spinal-evoked potentials in at least one muscle.

#### 2.2.3. Paired supraspinal and epidural SCS

Interaction between supraspinal inputs and epidural SCS was first assessed in two monkeys (monkeys Si and Un) by pairing suprathreshold ICMS (three biphasic pulses at 333 Hz, 0.1 ms per phase, cathodal first) with single pulse SCS, using interstimulus intervals between −100, −50, −40, −30, …, 40, 50,  100 ms after compensating for the longer response latency from ICMS so hand muscle responses coincided (both delivered using Model 2100 stimulators). Experiments were performed with an SCS intensity that was suprathreshold for activating a subset of muscles.

We next examined modulation of supraspinal responses during one second trains of subthreshold dorsal or ventral epidural SCS at frequencies of 10, 20, 50, 100, 143 or 200 Hz (monkeys Si and Un). ICMS was delivered either 500 ms before the beginning of the spinal train, between 0, 100, …, 1000 ms after the beginning of the train, or 500 ms after the end of the train, in pseudorandomized order.

In additional experiments (monkey Yi and Yu), similar stimulation protocols were repeated using single pulse pyramidal tract stimulation (PTS, biphasic, 0.1 ms per phase) in place of ICMS (figure 1(c)).

#### 2.2.4. Paired supraspinal and transcutaneous SCS

Trains of transcutaneous SCS was delivered using a Model 4100 stimulator (A-M Systems, Carlsborg, US). We used the ‘Russian current’ approach to delivering high currents without associated cutaneous discomfort, in which a high carrier frequency is modulated by a lower stimulation frequency. We tested carrier frequencies of 1, 2, 5 and 10 kHz, delivered as a burst of ten biphasic (0.05 ms per phase, cathodal first) pulses, with intervals of 1, 0.5, 0.2 and 0.1 ms respectively. We used stimulation frequencies of 10, 20, 50 and 100 Hz, i.e. with intervals of 100, 50, 20 and 10 ms between each burst of ten pulses. We delivered 1 s trains of this transcutaneous SCS pattern while testing modulation of ICMS responses as described above (figure 1(c)).

### 2.3. Analysis methods

#### 2.3.1. Intensity series

Following the method of Sharpe and Jackson (2014), EMG responses were rectified and averaged over a 10 ms post-stimulus window adapted to their latencies, and compared against an equivalent pre-stimulus time-window using a two-tailed unpaired *t*-test (*p* < 0.05) to construct recruitment curves, $R\left( I \right)$, quantifying the percentage of ipsilateral muscles exhibiting a significant response to each stimulus intensity, $I$. We then performed a least-squares fit to these curves with a cumulative Normal distribution:

1 \begin{equation*}R\left( I \right) = 100\% \times \int\limits_{ - \infty }^I N \left( {x - \mu ,{\sigma ^2}} \right){\text{d}}x\end{equation*}

to obtain estimates of the mean, $\mu $, and variance, ${\sigma ^2}$ of the response threshold across muscles. Note that this approach assumes thresholds are normally distributed, but has the advantage that mean thresholds can be assessed across different intensity ranges and even if not all muscles are activated by the highest intensity.

#### 2.3.2. Frequency series

EMG responses following each pulse of the SCS train were rectified and averaged over an appropriate 8 ms post-stimulus window (to allow exclusion of stimulus artefacts with the highest frequency trains). For muscles exhibiting a significant response to the first stimulus pulse, the response to each subsequent pulse in the train was normalized by the magnitude of the first response. These normalized values were then averaged across responding muscles to assess facilitation and suppression of subsequent responses during SCS trains of different frequencies.

#### 2.3.3. Paired supraspinal and spinal stimulation

In order to assess the impact of SCS on supraspinal-evoked responses, we compared the EMG elicited by paired stimulation to that predicted by a linear sum of responses to spinal and supraspinal stimuli delivered alone. Since rectification introduces a non-linearity, the average rectified responses cannot be added to generate this prediction. Instead, we used the method of (Baker and Lemon 1995) to generate a set of surrogate paired responses based on the linear summation of individual unrectified EMG responses with the appropriate interstimulus intervals, prior to rectification and averaging (see also Sharpe and Jackson 2014). Since these surrogates contain the summation of two sections of background activity (in addition to the evoked responses), we added a section of background activity (taken from a period without stimulation) to each response to paired stimulation before rectification and averaging. We used this method even when SCS appeared subthreshold to ensure that any occasional spinal-evoked potentials could not account for an increase in the paired response. We quantified modulation as the percentage ratio of the paired response to that predicted from linear summation. Thus a modulation >100% represents facilitation of the supraspinal response and a modulation <100% represents suppression.

To test whether modulation depended on SCS frequency, we averaged values across all interstimulus intervals within the train, and across all muscles responding to supraspinal stimulation alone. We then constructed 10 000 surrogate datasets in which we shuffled the responses for each interstimulus interval across stimulation frequencies. We used the variance of the modulation (across frequencies) as a test statistic and compared this with the 95th percentile of the null distribution obtained from the shuffled surrogates.

#### 2.3.4. Modelling muscles responses to spinal and supraspinal stimulation

We have previously used a simple computational model of combined excitatory and inhibitory influences (Zimmermann *et al* 2011, Sharpe and Jackson 2014) to explain the frequency-dependent modulation of responses to SCS trains. We adapted this model to simulate also the modulation of supraspinal inputs. This was not intended as a detailed biophysical simulation, but an attempt to capture the qualitative features of our results with minimal free parameters. Briefly, our original model assumed that preceding pulses in a SCS train act both to facilitate (with a short decay time) and suppress (with a longer decay) the responses to subsequent stimuli. In the present work, we first assumed that this was mediated by the accumulation of post-synaptic potentials within the membrane potential of a population of neurons, ${V_{{\text{mem}}}}$, with arbitrary units. In our first model, this was given by:

2 \begin{align*}&amp;{V_{{\text{mem}}}}\left( t \right)\nonumber\\ &amp;\quad = {V_{{\text{rest}}}} + {\text{ }}\sum\limits_n {\left[ {{A_{\text{f}}}{\text{P}}\left( {\frac{{t - {t_n}}}{{{\tau _{\text{f}}}}}} \right) - {A_{\text{s}}}{\text{P}}\left( {\frac{{t - {t_n}}}{{{\tau _{\text{s}}}}}} \right)} \right]} \end{align*}

where ${V_{{\text{rest}}}}$ represents the negative baseline resting membrane potential, ${A_{\text{f}}}$ and ${A_{\text{s}}}$ represent the strengths of facilitation and suppression, and ${\tau _{\text{f}}}$ and ${\tau _{\text{s}}}$ represent their respective time constants. ${t_n}$ are the times of 15 equally-spaced stimulus pulses, and $P\left( x \right)$ is an exponentially-decaying synaptic potential:

3 \begin{equation*}P\left( x \right) = \left\{ {\begin{array}{*{20}{l}} {{e^{ - x}}\kern1.5pt{\text{ for }}\kern1.5ptx \geqslant 0} \\ {0\kern1.5pt{\text{ for }}\kern1.5ptx &lt; 0} \end{array}} \right\}.\end{equation*}

In our second model, we assumed that suppression did not act directly on the motoneurons, but instead via an upstream mechanism that gated the facilitation that reached the motoneurons, for example via primary afferent depolarisation (PAD). We modelled afferent depolarisation, ${V_{aff}}$ as:

4 \begin{equation*}{V_{{\text{aff}}}}\left( t \right) = {\text{ }}\sum\limits_n {{A_{\text{s}}}} {\text{P}}\left( {\frac{{t - {t_n}}}{{{\tau _{\text{s}}}}}} \right)\end{equation*}

and assumed this gated the excitatory post-synaptic potentials in motoneurons according to:

5 \begin{equation*}{V_{{\text{mem}}}}\left( t \right) = {V_{{\text{rest}}}} + {\text{ }}\sum\limits_n {{A_{\text{f}}}} P\left( {{V_{{\text{aff}}}}\left( {{t_n}} \right)} \right){\text{P}}\left( {\frac{{t - {t_n}}}{{{\tau _{\text{f}}}}}} \right).\end{equation*}

Note that in the first model (equation (2)), ${V_{{\text{mem}}}}$ becomes progressively negative during the SCS train as the slowly decaying inhibitory potentials accumulate. In the second model (equation (5)), ${V_{{\text{mem}}}}$ instead decays towards the base-line resting potential as the facilitatory influence of SCS is gated upstream. This leads to a key difference between model predictions for responses to supraspinal stimulation during SCS trains. In the first model, muscles become unresponsive to both spinal and supraspinal stimuli due to direct inhibition of motoneurons. By contrast, in the second model, motoneurons become unresponsive to subsequent spinal stimuli (due to gating of afferent input) but still respond to supraspinal inputs at close to baseline levels.

To convert the membrane potential into a response to stimulation, we assumed that the firing threshold of the motoneuron populations was normally distributed with zero mean and unity variance (these arbitrary scaling factors are accommodated by the other model parameters). Thus the proportion of motoneurons responding to stimulation was equal to:

6 \begin{equation*}S\left( {{V_{{\text{mem}}}}} \right) = {\text{ }}\int\limits_{ - {V_{{\text{mem}}}}}^\infty N \left( {0,1} \right){\text{ d}}x\end{equation*}

and the modulation of that response relative to baseline was equal to:

7 \begin{equation*}{M_n} = 100\% \times \frac{{S\left( {{V_{{\text{mem}}}}\left( {{t_n}} \right)} \right)}}{{S\left( {{V_{{\text{mem}}}}\left( {{t_1}} \right)} \right)}}.\end{equation*}

Each model comprised five free parameters: the strengths of facilitation, ${A_{\text{f}}}$, and suppression, ${A_{\text{s}}}$, their respective time-constants, ${\tau _{\text{f}}}$ and ${\tau _{\text{s}}}$, which govern the temporal modulation of responses, and the resting motoneuron potential, ${V_{{\text{rest}}}}$ which affects only their overall magnitude. We chose ${V_{{\text{rest}}}}$ such that 50% of motoneurons responded to the first stimulus in the train (i.e. such that $S\left( {{V_{{\text{mem}}}}\left( {{t_1}} \right)} \right) = 0.5$). We then fit the remaining four parameters to the experimentally-observed temporal modulation of responses to dorsal spinal stimulation trains at different frequencies using least-squares regression. We fit the first five responses in each train, and assessed model fit over the same data using a coefficient of determination:

8 \begin{equation*}{R^2} = 1 - \frac{{{\text{Var}}\left( {{M_{{\text{fit}}}} - {M_{{\text{actual}}}}} \right)}}{{{\text{Var}}\left( {{M_{{\text{actual}}}}} \right)}}.\end{equation*}

Finally, we used each model to predict the modulation of weak supraspinal inputs, based on the parameters fit to responses to spinal stimulation alone. We first calculated the membrane potential resulting from 1 s trains of spinal stimulation at different frequencies (using equations (2) or (5)). We assumed that the supraspinal inputs would be dispersed in time, so we used the average value of ${V_{{\text{mem}}}}$ across consecutive 100 ms epochs through the train. We then used equations (6) and (7) to calculate the modulation of a weak supraspinal stimulus assumed to recruit 1% of motoneurons at baseline (i.e. such that $S\left( {{V_{{\text{rest}}}}} \right) = 0.01$).

All analyses were performed using custom software written in Matlab (Mathworks, Natick, US).

## 3. Results

### 3.1. Ventral epidural SCS effectively recruits upper-limb muscles

**Figure 2.**
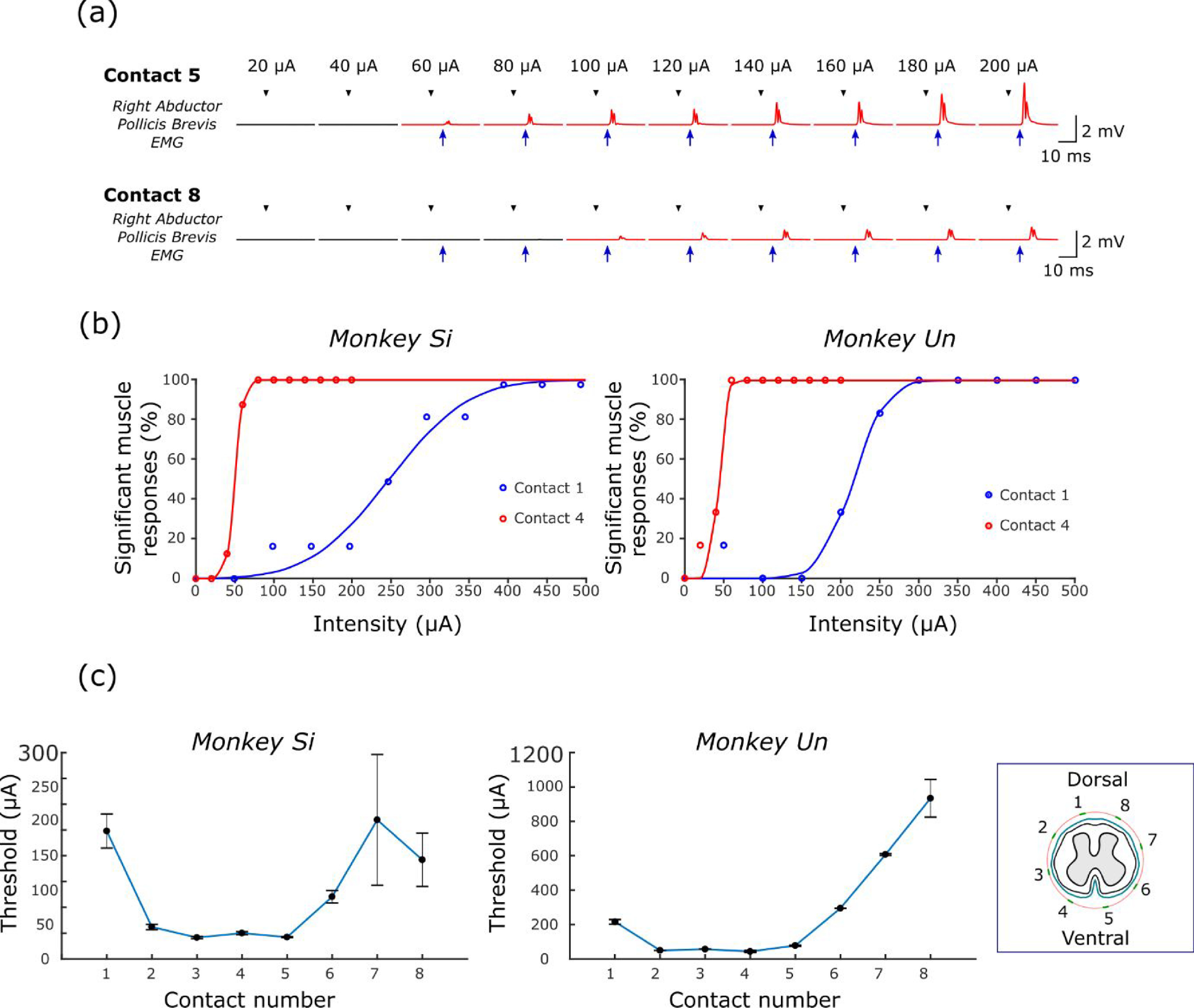
Intensity series results. (a) Example mean, rectified EMG responses in right abductor pollicis brevis (APB) muscle elicited by single-pulse ventral (contact 5) and dorsal (contact 8) epidural SCS with intensities between 20 and 200 *μ*A in monkey Si. Red traces indicate statistically-significant response (paired *t*-test, *P* < 0.05). Black triangles indicate time of stimulation while blue arrows indicate response onset. (b) Proportion of ipsilateral muscles exhibiting significant responses to single-pulse ventral (contact 4) and dorsal (contact 1) SCS. Data are fit with a cumulative normal distribution to assess average activation threshold. (c) Average activation thresholds for all electrode contacts. The lowest thresholds are obtained for contacts on the ventral side of the spinal cord. Error bars indicate s.e.m over the six ipsilateral muscles.

We first characterized responses in upper-limb muscle to epidural SCS delivered through different contacts on the epidural spinal array. Spinal-evoked potentials were quantified by average rectified EMG, and compared to a pre-stimulus baseline period to assess statistical significance. Figure 2(a) shows example responses in a single muscle to stimulation delivered at 20–200 *μ*A through dorsal and ventral contacts. Note that muscle responses to dorsal stimulation occurred with longer latency and higher stimulus intensities compared to ventral stimulation. Figure 2(b) shows the proportion of all recorded muscles exhibiting a significant evoked potential at different stimulus intensities. We fitted these recruitment curves with cumulative Gaussian distributions to obtain average response thresholds. Response thresholds were lowest for contacts on the ventral side of the spinal cord (typically below 50 *μ*A), and higher for contacts on the dorsal side (typically greater than 200 *μ*A) (figure 2(c)). A circular-linear correlation analysis showed a significant relationship between the positioning of the contact around the spinal cord and thresholds of muscle activation (circular-linear correlation *R* = 0.92, *P* = 0.035).

**Figure 3.**
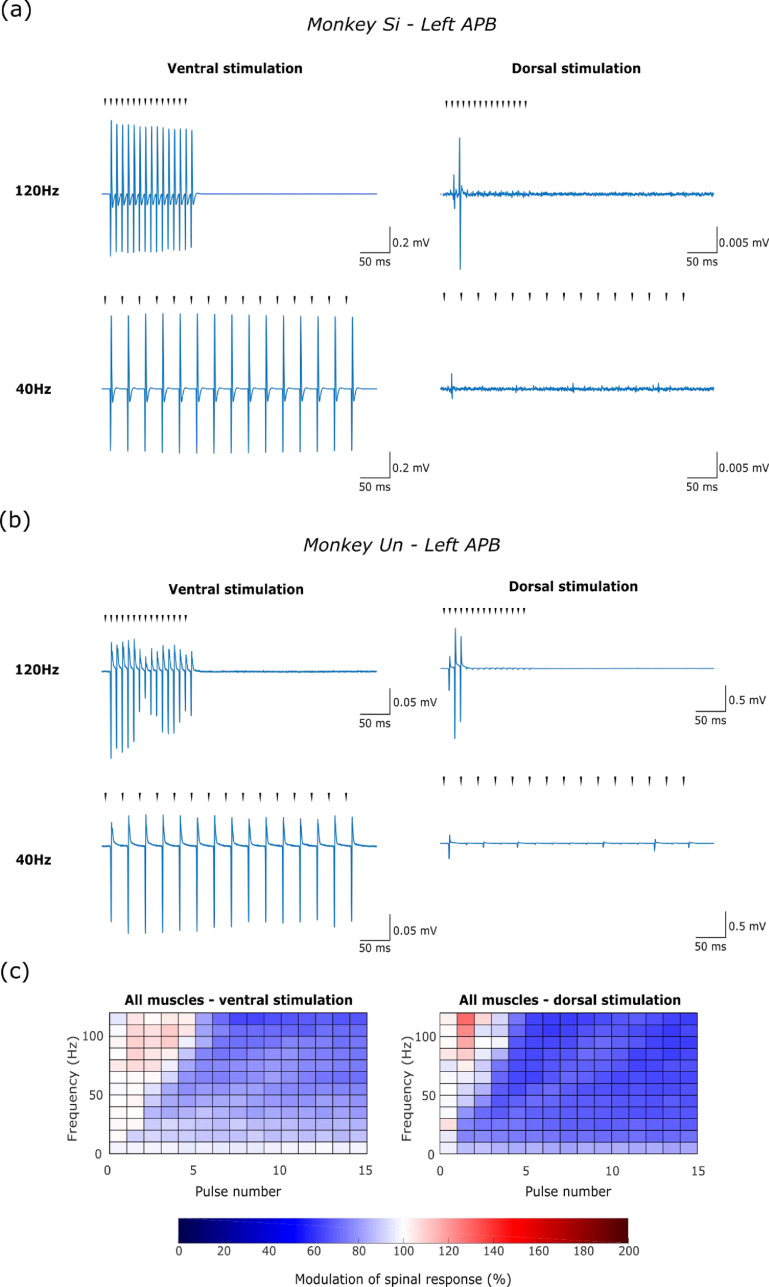
Frequency series results. (a) Example EMG responses in left APB elicited by trains of ventral (contact 3—left panel) and dorsal (contact 1—right panel) SCS at 120 Hz (top) and 40 Hz (bottom) in monkey Si. Triangles indicate times of stimuli. (b) Similar data for left APB responses in monkey Un. (c) Modulation of mean, rectified EMG response to each pulse in the train, normalized by the response to the first pulse, averaged across all muscles responding to ventral (left panel—24 muscles) and dorsal (right panel—22 muscles) SCS in monkeys Si and Un.

In addition, we examined the reliability of muscle responses evoked by stimulus trains with different frequencies between 10 and 120 Hz. For ventral stimulation, consistent responses could be observed following each stimulus even at the highest frequencies (figures 3(a) and (b); left column). By contrast, when delivering high-frequency stimulation to the dorsal surface, the response to the second and third pulses in the train was often facilitated relative to the first pulse, while responses to later stimuli were profoundly suppressed (figures 3(a) and (b); right column). Figure 3(c) summarizes modulation of responses to both ventral and dorsal stimulation for each pulse within the trains of different frequencies.

These findings are consistent with the results of Sharpe and Jackson (2014), and suggest that stimulation of the ventral surface acts directly on the axons of the motoneurons, while stimulation of the dorsal surface activates motoneurons transynaptically, leading to higher recruitment thresholds and temporal facilitation/suppression of subsequent responses.

### 3.2. Single-pulse dorsal epidural SCS facilitates cortical-evoked responses

**Figure 4.**
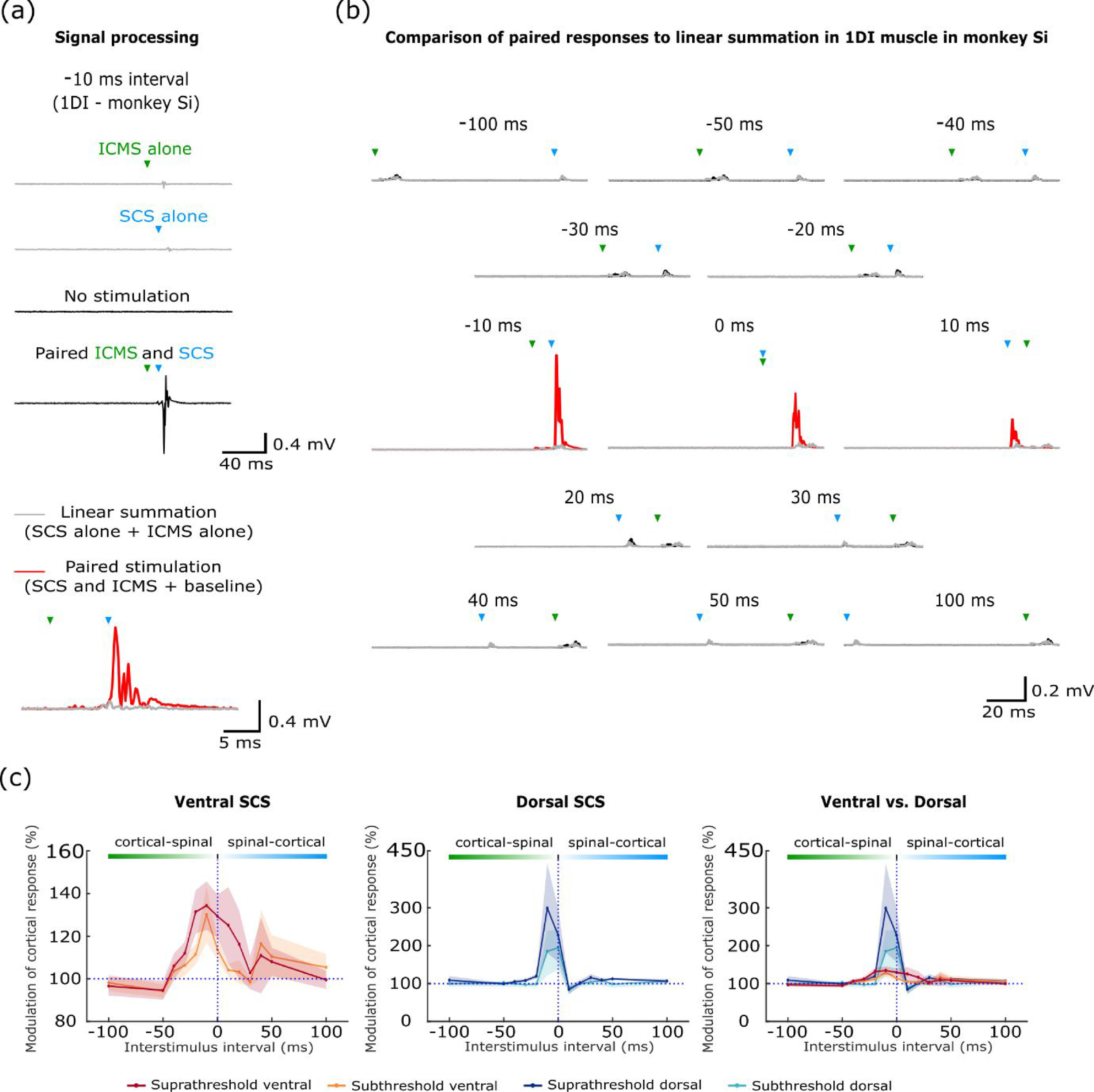
Non-linear interactions between cortical- and spinal-evoked muscle responses. (a) EMG responses to paired ICMS and epidural SCS were compared to surrogate data generated from the linear summation of responses to each stimulus alone. Prior to rectification and averaging, a baseline trace with no stimulation was added to the paired response to compensate for the higher background level in the surrogate traces. This example shows supralinear facilitation of the response in 1DI muscle in monkey Si when ICMS preceded SCS by 10 ms in monkey Si. Green and blue triangles correspond to cortical and spinal stimulation respectively. (b) Comparison of paired responses to linear summation for all interstimulus intervals between −100 and 100 ms. Red/black traces indicate significant/non-significant supralinear facilitation (paired *t*-test against linear summation surrogates, grey traces, *P* < 0.05). (c) Modulation of response to paired stimulation relative to linear summation for all muscles that responded to ICMS in monkeys Si and Un. Muscles are divided according to whether the spinal stimulus alone was suprathreshold or subthreshold for eliciting a response. Left panel shows results for ventral epidural SCS (seven muscles). Middle panel shows results for dorsal epidural SCS (five muscles). Right panel compares modulation for dorsal vs. ventral epidural SCS, on the same ordinate scale. Shading indicates s.e.m over muscle-electrode pairs.

Next, we examined interactions between single pulse SCS and descending volleys elicited by ICMS. We used interstimulus intervals between −100 and +100 ms, adjusted according to the different conduction times from cortex and spinal cord to the periphery. Mean rectified EMG was calculated over a response window following the second of the two stimuli, and compared to that predicted by a linear superposition of the response to each stimulus alone (figures 4(a) and (b)). We used a spinal stimulation intensity that activated around half of the recorded muscles, and assessed separately ICMS responses in muscles for which spinal stimulation was below or above threshold (figure 4(c)). Only weak facilitation of the cortical response was observed for both suprathreshold (mean${\text{ }}[$standard deviation] facilitation of 134% [11%]) and subthreshold (130% [13%]) ventral SCS (preceding the cortical stimulus by 10 ms). By contrast, dorsal SCS was more effective at potentiating descending inputs, with mean (SD) peak facilitations of 298% (120%) for suprathreshold stimuli and 248% (54%) for subthreshold stimuli. The difference between dorsal and ventral stimulation was statistically significant (10 ms interval, paired t-test across responding muscles, *t*_9_ = 1.96, *P* = 0.041), suggesting that transynaptic activation of motoneurons from the dorsal surface, but not direct activation from the ventral surface, is most effective at facilitating the response to descending inputs.

### 3.3. Trains of dorsal epidural SCS facilitate supraspinal inputs in a frequency-dependent manner

**Figure 5.**
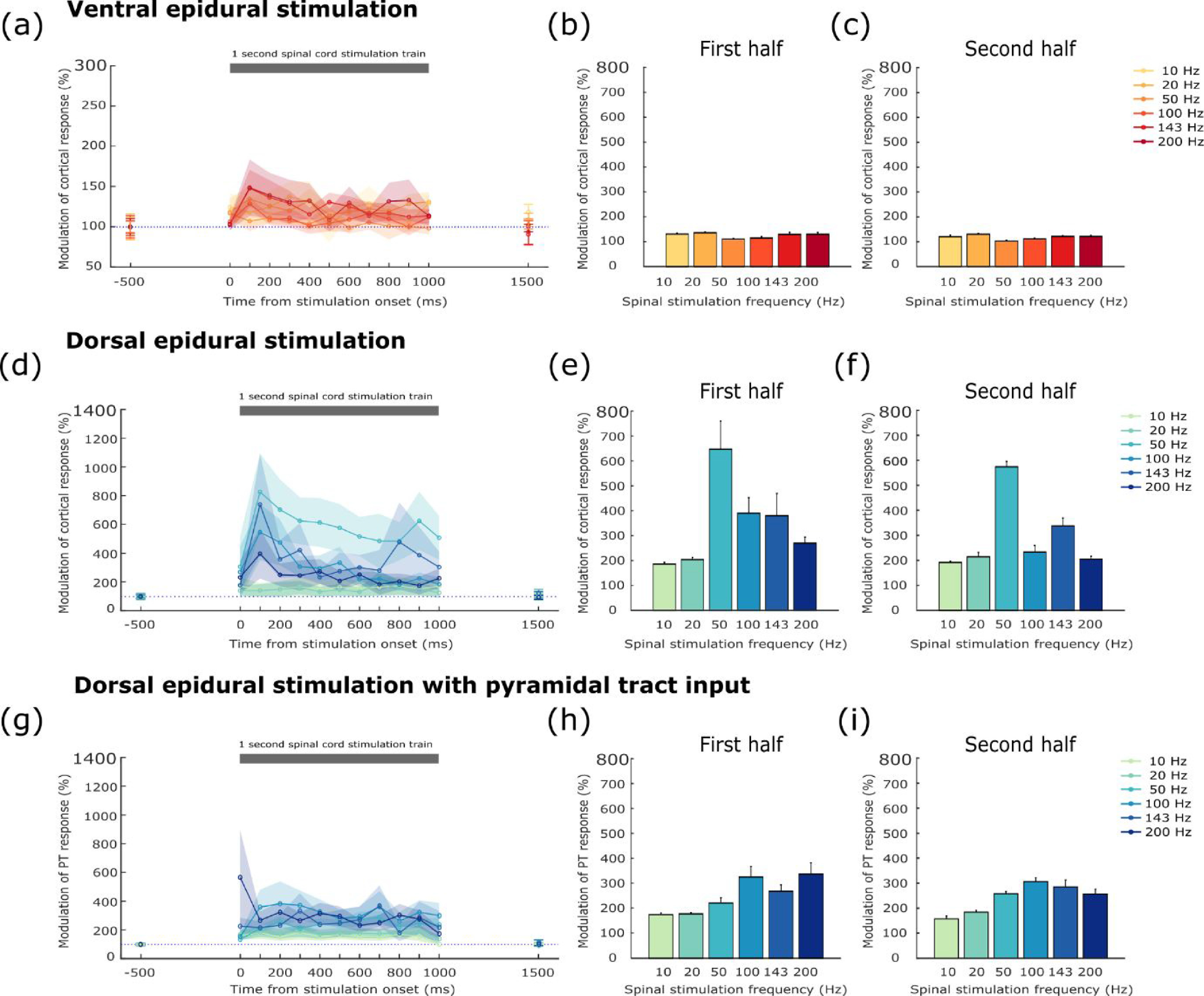
Facilitation of supraspinal-evoked muscle responses by trains of epidural SCS. (a) Modulation of EMG responses to ICMS delivered at different times during a 1 s train of ventral epidural SCS. Colours indicate different frequencies of spinal stimulation. Data from four muscle-electrode pairs in monkeys Si and Un. Shading indicates s.e.m over muscle-electrode pairs. (b), (c) Mean modulation of cortical-evoked responses during the first and second halves of the spinal train respectively. Error bars indicate s.e.m over intervals (d), (e), (f) Equivalent plots for dorsal epidural SCS. Data from four muscle-electrode pairs in monkeys Si and Un. (g), (h), (i) Equivalent plots for modulation of EMG responses to PT stimulation during dorsal epidural SCS. Data from ten muscle-electrode pairs in monkeys Yu and Yi.

We examined the temporal profile of facilitation by 1 s trains of subthreshold SCS delivered at frequencies from 10 to 200 Hz. On separate trials, ICMS was delivered at different time-points to probe excitability changes through the train. Figure 5 (left column) shows modulation of the cortical-evoked potential (expressed as a percentage of the linear superposition of cortical and spinal responses) for all time intervals, while the histograms show the average modulation over the first (middle) or second (right) halves of the train.

**Figure 6.**
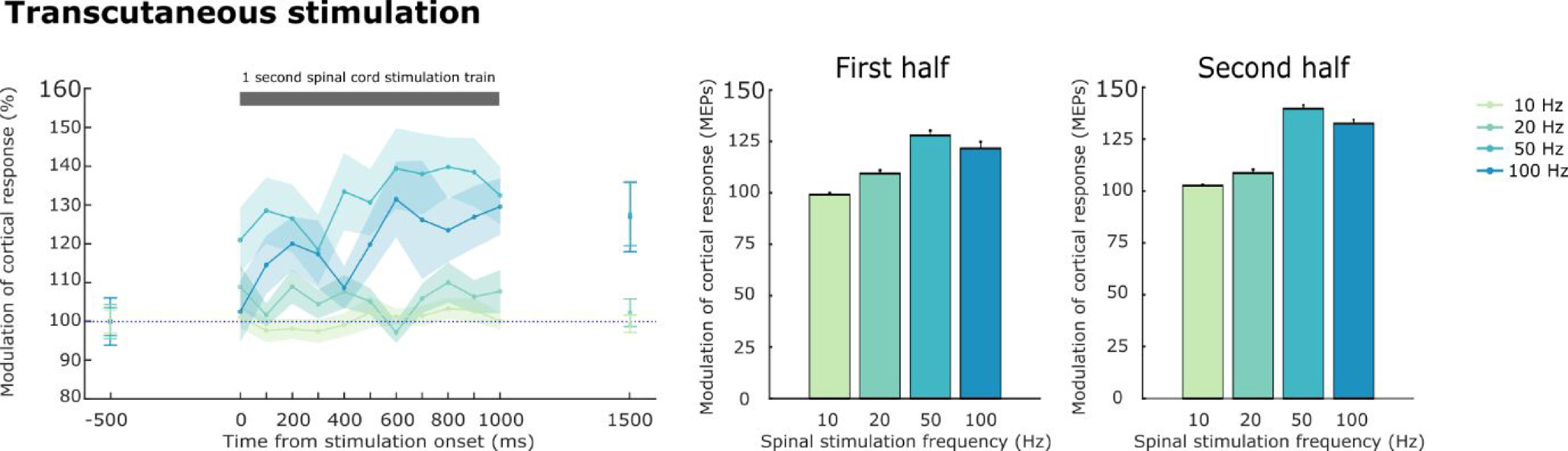
Facilitation of cortical-evoked muscle responses by trains of transcutaneous SCS (5 kHz carrier frequency). Left panel shows modulation of EMG responses to ICMS delivered at different times during a 1 s train of transcutaneous SCS. Colours indicate different frequencies of spinal stimulation. Shading indicates s.e.m. over muscle-electrode pairs. Data from 27 muscle-electrode pairs in monkeys Uk and Yu. Middle and right panels show mean modulation of cortical-evoked response during the first and second halves of the spinal train respectively. Error bars indicate s.e.m. over intervals.

In general, cortical-evoked potentials were facilitated when delivered during the SCS train, with responses induced by paired stimulation being the same or higher (>100%) than that predicted by linear superposition. Facilitation was significantly greater for SCS delivered to the dorsal surface (mean [SD] facilitation of 316% [137%]; figures 6(d), (e) and (f)) compared to ventral stimulation (121% [23%]; figures 6(a), (b) and (c); paired *t*-test across all responsive muscles, *t*_3_ = 2.64, *P* = 0.039) There was a general trend for facilitation to decrease through the train, especially for high frequencies of SCS. Thus, facilitation during the first half of the dorsal epidural SCS train (345% [150%]; figure 5(e)) was significantly higher than during the second half (291% [128%]; figure 5(f); paired *t*-test across responsive muscles, *t*_3_ = 3.06, *P* = 0.028).

We used a shuffling analysis to test the influence of spinal stimulation frequency (see section 2). This revealed a statistically significant (*P* < 0.05) effect of frequency for both ventral (${\text{Var}}\left( {{\text{Data}}} \right) = 92.2,{\text{ Var}}\left( {95\% {\text{ Shuffled}}} \right) = 58.5$) and dorsal epidural SCS (${\text{Var}}\left( {{\text{Data}}} \right) = 2.39$ × ${10^4}$, ${\text{Var}}\left( {95\% {\text{Shuffled}}} \right) = 6.32$ × ${10^3}$). For ventral stimulation, the greatest facilitation was seen with the highest stimulation frequency of 200 Hz (132% [42%]). By contrast, for dorsal stimulation, an intermediate frequency of 50 Hz was optimal (605% [391%]).

To demonstrate that facilitation occurred at the spinal level, rather than as a result solely of afferent volleys increasing cortical excitability, we also tested facilitation of motor responses elicited by PTS. Figures 5(g), (h) and (i) shows that facilitation of PT-evoked potentials could be obtained with dorsal epidural SCS, with muscle responses exhibiting facilitation (>100%) relative to linear summation throughout the SCS train. PTS responses were facilitated less than cortical-evoked potentials when paired with dorsal SCS (245% [115%] vs. 316% [137%]) but these recordings were made in different animals and this difference in any case did not reach significance (unpaired *t*-test across responsive muscles, *t*_12_ = 0.92, *P* = 0.2). The greatest facilitation was again obtained for an intermediate stimulation frequencies, with a maximum facilitation of 314% (252%) when stimulating at 100 Hz. Again, our shuffling analysis revealed a significant impact of SCS frequency on facilitation (${\text{Var}}\left( {{\text{Data}}} \right) = 3.71$ ×${10^3},{\text{ Var}}\left( {95\% {\text{Shuffled}}} \right) = 1.79$ × ${10^3}$).

We conclude that trains of subthreshold epidural SCS delivered to the dorsal aspect of the spinal cord are effective at facilitating the muscle response to supraspinal inputs. This is likely mediated by transynaptic inputs to motoneurons that raise their membrane potential closer to threshold, making them more responsive to descending volleys. Moreover, this effect is frequency-dependent, such that intermediate frequencies (50 or 100 Hz) are more effective than either lower or higher frequencies of stimulation.

### 3.4. Facilitation of descending pathways by transcutaneous SCS

Our final set of experiments sought to establish whether similar facilitation of supraspinal inputs could be achieved using transcutaneous SCS. We delivered 1 s trains of ‘Russian current’ stimulation (low-frequency modulation of a high-frequency carrier) through surface electrodes placed on the skin over the upper back (figure 6). Again we observed facilitation of ICMS responses, although the magnitude of facilitation was lower than that obtained with epidural stimulation. As with dorsal epidural SCS, the most effective modulation frequency was 50 Hz, (mean [SD] facilitation of 134% [39%]), and our shuffling method again verified a significant impact of transcutaneous stimulation frequency (${\text{Var}}\left( {{\text{Data}}} \right) = 242,{\text{ Var}}\left( {95\% {\text{Shuffled}}} \right) = 13$). Unlike epidural SCS, the effect of transcutaneous SCS grew throughout the train (115% [22%] and 121% [22%] for first and second halves respectively; paired *t*-test *t*_26_ = 3.38, *P* = 0.001). Moreover, facilitation outlasted the end of the train, with significantly enhanced responses occurring 500 ms after the train compared to 500 ms before (118% [18%], paired *t*-test *t*_26_ = 4.71, *P* = 3.6 × 10^−5^).

**Figure 7.**
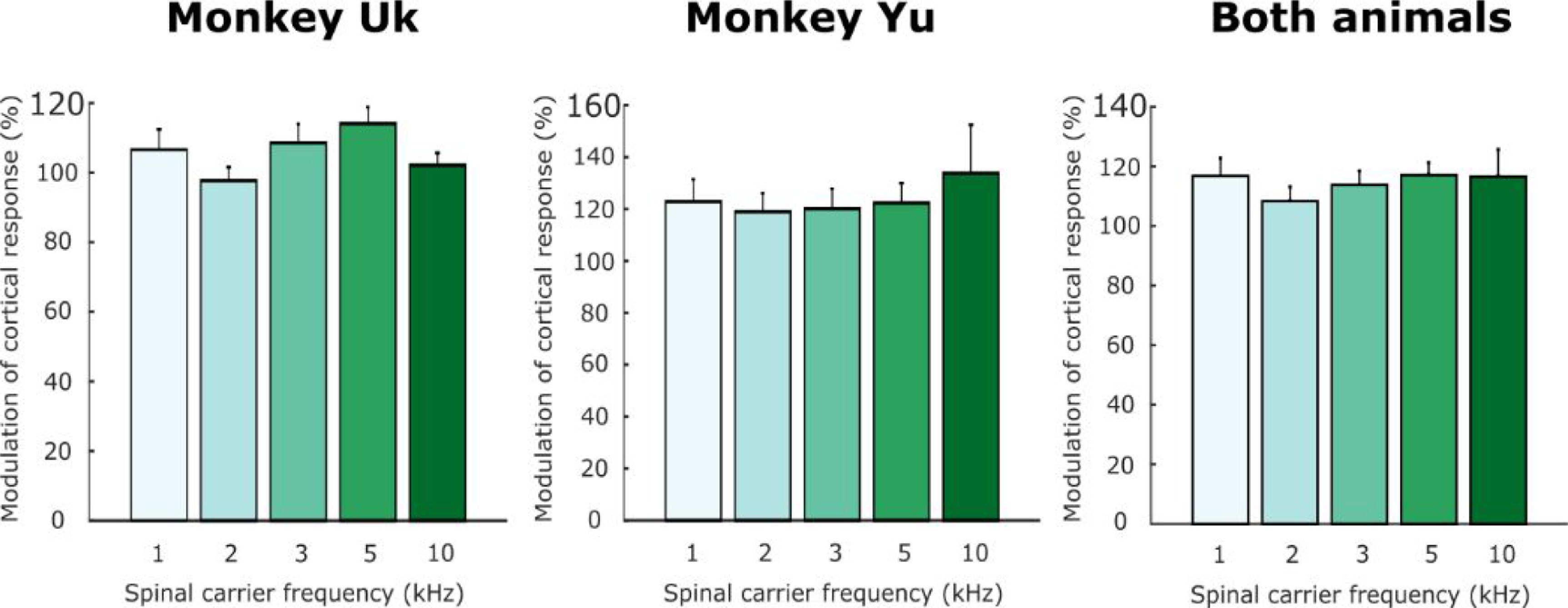
Effect of different carrier frequencies for transcutaneous SCS. Plots show mean modulation of cortical-evoked responses during trains of transcutaneous SCS at 50 Hz with different carrier frequencies in monkeys Uk and Yu. Data from 27 muscle-electrode pairs in monkeys Uk and Yu. Error bars indicate s.e.m. over intervals.

These experiments were conducted using a carrier frequency of 5 kHz, based on pilot experiments in our first animal (monkey Uk, three sessions, figure 7). We initially compared five different carrier frequencies embedded in a 50 Hz stimulation pattern (1 kHz, 2 kHz, 3 kHz, 5 kHz and 10 kHz) and found that 5 kHz provided the best facilitation (114% [20%]), although a single factor analysis of variance (ANOVA) failed to show a significant impact of carrying frequencies on facilitation (single factor ANOVA, ${F_{4,57}} = 1.97,{\text{ }}P = 0.11$). We subsequently examined the effect of carrier frequency in our second animal (Monkey Yu, four sessions) and combining data from both animals confirmed the absence of significant effect of carrier frequency (${F_{4,107}} = 0.37,{\text{ }}P = 0.83$).

### 3.5. Modelling spinal modulation as synaptic excitation gated by upstream inhibition

**Figure 8.**
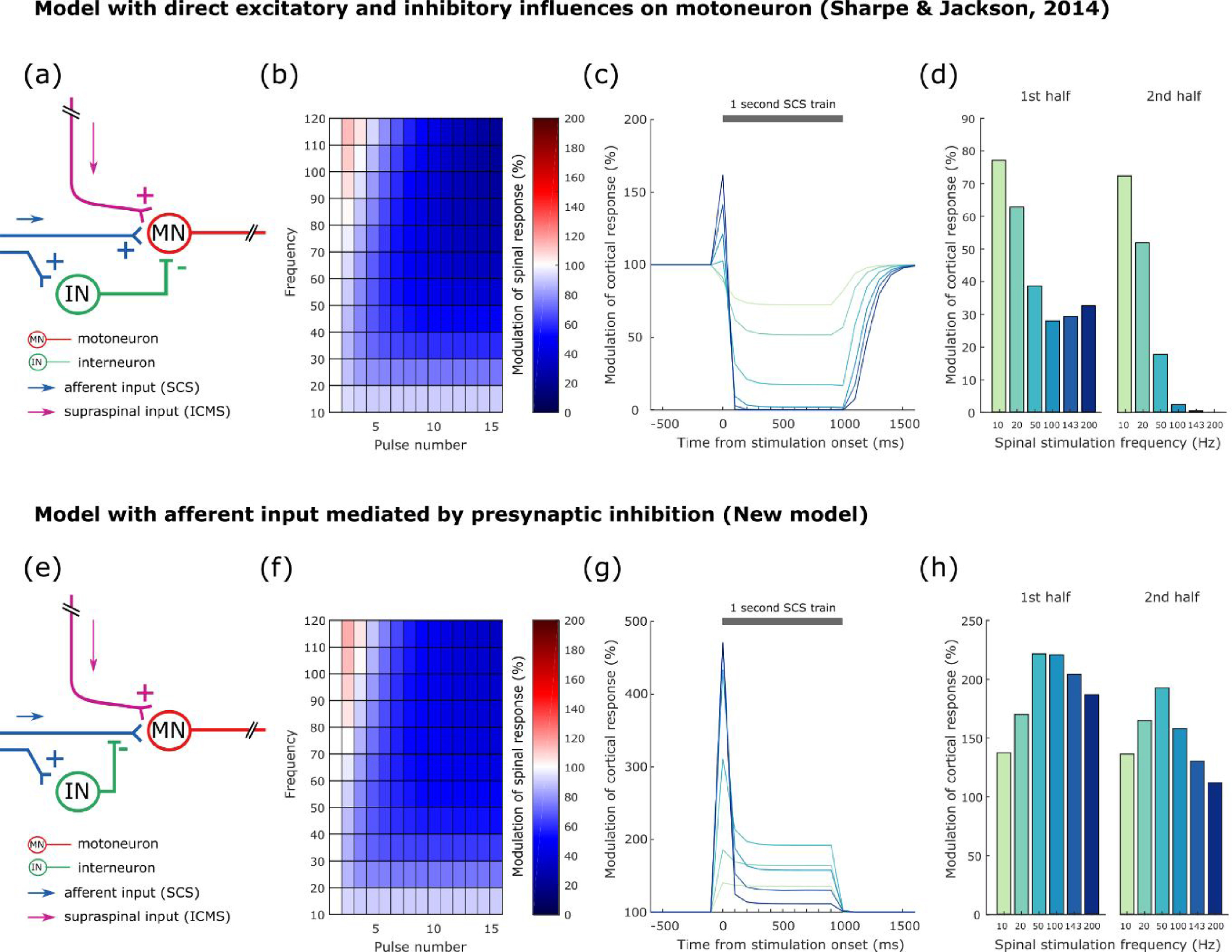
Computational model of time- and frequency-dependence of SCS effects. (a) Our first model assumed that excitatory and inhibitory influences of SCS (decaying over different time-scales) accrue at the motoneurons. (b) The model reproduced the experimentally-observed modulation of spinal-evoked responses to different frequencies of SCS (figure 3(c)) with a least-squares fit, *R*^2^ = 0.87. (c), (d) The same model and fit parameters were then used to predict modulation of cortical-evoked responses by SCS. This yielded suppression of cortical-evoked responses, especially for high-frequency SCS, due to accumulation of inhibition on motoneurons, in contrast to experimental results. (e) Our second model assumed inhibition acted upstream of the motoneurons to gate the excitatory influence, for example via presynaptic inhibition. (f) This model could also reproduce the modulation of spinal-evoked responses with a least-squares fit, *R*^2^ = 0.90. (g), (h) This model could qualitatively reproduce the experimental results of modulation of cortical-evoked responses by SCS, predicting facilitation, especially for intermediate SCS frequencies.

Previously we have introduced a simple mathematical description of the frequency-dependence of muscle responses to SCS trains (Zimmerman *et al* 2011, Sharpe and Jackson 2014). Briefly, we assumed that the response to each pulse in the train was modulated by an accumulation of excitatory and inhibitory influences from preceding stimulus pulses. By modelling the decay of these influences as exponentials with fast and slow time-courses respectively, we could explain the transient facilitation of responses to high-frequency SCS and later response suppression for high- and intermediate-frequency trains. While not intended to be a realistic biophysical model of spinal circuits, we were interested in whether this same description could explain the frequency-dependent modulation of supraspinal inputs in our current dataset. Our first attempt assumed the excitatory and inhibitory influences of spinal stimulation both acted to raise or lower the membrane threshold of a population of spinal motoneurons (figure 8(a)). As a result, different stimuli within the train recruited a varying proportion of the population. We used least-squares regression to fit the experimentally-observed modulation of responses to suprathreshold dorsal epidural SCS (figure 3(c), left), obtaining time-constants for facilitation and suppression of 7 ms and 87 ms respectively. The model reproduced the time- and frequency-dependence of these responses well (figure 8(b)), with a coefficient of determination (*R*^2^) of 0.87. However, it failed to replicate the experimental results for modulation of ICMS responses (figures 8(c) and (d)). In particular, the model predicted strong suppression of ICMS responses during the later period of high-frequency spinal stimulation, as the slowly-decaying inhibition builds up. This highlights a puzzling qualitative feature of our results. While high-frequency SCS inhibits the response to subsequent spinal stimuli (i.e. to less than 100% of the response to the first stimulus), it merely reduces the facilitation of supraspinal inputs (i.e. declining towards 100% of the response to ICMS alone).

Therefore we speculated that the inhibitory effect of SCS may not act directly on the motoneurons but upstream, gating subsequent spinal but not supraspinal inputs (figure 8(e)). A possible mechanism for such gating would be presynaptic inhibition via PAD. We modified our original model such that the inhibitory influence of preceding stimulus pulses modulated the extent to which subsequent SCS pulses excited the population of motoneurons (see section 2). This model was again capable of reproducing the frequency-dependence of motor responses to SCS alone (figure 8(f)), with similar best-fit time-constants for facilitation and suppression (9 ms and 74 ms respectively) and *R*^2^ = 0.90. However, the new model was also able to qualitatively capture the modulation of ICMS responses by SCS trains (figures 8(g) and (h)). For low SCS frequencies (10–50 Hz), the influence of preceding stimuli was small, and thus the excitatory effects produced a facilitation of the ICMS response that became progressively larger with increasing frequency of pulses. However, as SCS frequency increased further (100–200 Hz), the inhibitory influence of previous pulses diminished this excitatory effect, particularly during the latter part of the train. As a result, modulation of ICMS responses was greatest for an intermediate frequency (50 Hz) but never reduced below 100%, as in the experimental data.

## 4. Discussion

We investigated whether SCS delivered via ventral epidural, dorsal epidural and dorsal transcutaneous electrodes could modulate muscle responses evoked from descending pathways. The largest facilitation was observed using dorsal epidural SCS at an intermediate stimulation frequency of 50 Hz, which increased the magnitude of EMG responses to ICMS by around six times. This was likely driven largely by an interaction at the spinal level, as comparable facilitation was also observed in motor responses to pyramidal tract stimulation which elicits a constant descending volley unaffected by modulations in cortical excitability. These results support the idea that subthreshold epidural SCS may be beneficial in the case of anatomically incomplete spinal cord injuries, by transynaptically increasing the excitability of the spinal cord and thus facilitating volitional control of muscles mediated by surviving descending connections.

By contrast, ventral epidural SCS produced considerably less modulation of muscle responses to descending volleys. However, suprathreshold ventral epidural SCS was more effective at driving muscles, with lower thresholds and EMG responses that consistently followed stimulation pulses even with high-frequency trains. This is consistent with a direct action on the motor unit, for example, eliciting action potentials in the ventral roots. Thus ventral epidural SCS may be well-suited for strong, direct activation of paralyzed muscles for FES applications. The flexible cuff electrode we used in this study allowed contacts to be placed on both dorsal and ventral surfaces, and could in future be adapted for human use, such that both types of stimulation might be delivered in combination as appropriate for patient needs. Our experiments were performed using neurologically intact animals under anaesthesia so it remains to be seen if similar results can be obtained in awake, spinal cord-injured subjects. However, dorsal SCS in lumbar segments has already proved effective in human individuals (Harkema *et al* 2011, Angeli *et al* 2018), while the direct action of ventral SCS on motor units should not be greatly influenced by upstream reorganization of spinal circuitry.

We previously compared ventral and dorsal epidural SCS using a ball electrode placed on the dura (Sharpe and Jackson 2014). One discrepancy worth noting is that in our previous study, thresholds for eliciting movements with epidural SCS were puzzlingly higher on the ventral vs. dorsal side of the cord. This was not the case in the present study using the cuff electrode. We do not have a definitive explanation for this, but our previous surgical approach involved accessing the spinal cord through the vertebral body, which was surgically awkward and may have contributed to either damage to the spinal cord or inaccurate positioning of electrodes on the ventral side. By contrast, the epidural cuff electrode provided efficient stimulation of the ventral spinal cord, and could be placed with relative ease via a dorsal laminectomy. In future it may be possible to reduce the need for an extensive laminectomy using the techniques of minimally-invasive spinal surgery (Vaishnav *et al* 2019).

We also found that transcutaneous SCS was capable of facilitating cortically-evoked muscle responses, although this was less effective than dorsal epidural SCS. Interestingly, and in contrast to epidural SCS, the degree of facilitation progressively increased during the 1 s stimulation trains, and was still evident 0.5 s after the end of the train. It is possible that transcutaneous SCS accesses additional spinal circuitry compared with epidural SCS, or even contributes to short-term plasticity mechanisms (Benavides *et al* 2020). However, it is worth noting that unlike with epidural SCS, we observed activation of back musculature under the electrodes during transcutaneous stimulation. Therefore it is likely that reafference or nociceptive effects could have contributed to the facilitatory effects of these stimulus trains. As with dorsal epidural SCS, facilitation was greatest with an intermediate stimulation frequency of 50 Hz.

Interestingly, these intermediate SCS frequencies around 50 Hz were not particularly effective at driving muscles directly. As in our previous studies, we found that muscles would follow trains of dorsal epidural SCS only up to around 10 Hz, above which responses to subsequent stimuli were progressively attenuated. At frequencies above 50 Hz, we observed facilitation of the first few responses followed by pronounced suppression. However, a simple computational model was able to reconcile these observations, by assuming that an excitatory synaptic influence of SCS on motoneurons was gated by upstream inhibition. Thus, for low rates, increasing the frequency of stimulation increased the net excitatory influence on motoneurons. However, beyond a certain point, the increased stimulation frequency attenuated the facilitation, and the excitability of motoneurons returned towards baseline. Our fit to the experimental data yielded excitatory decay-times of around 10 ms, close to the motoneuron membrane time-constant (Burke 1967). However, the inhibitory influence decayed with a much longer time-constant of 74 ms, which seems much too slow for inhibitory post-synaptic potentials. We suggest that the inhibitory influence may be mediated by presynaptic mechanisms, which have a similarly slow time-course (Eccles *et al* 1962, Jankowska *et al* 1981) and are known to act on afferents but not corticospinal inputs to motoneurons (Jackson *et al* 2006). Since dorsal SCS is thought to act primarily on the dorsal roots (Ladenbauer *et al* 2010), our data suggest that each spinal stimulus has at least two effects: first it produces a fast-decaying compound excitatory post-synaptic potential in the motoneurons (e.g. via Ia afferents) and, second, it produces slower presynaptic inhibition (via GABAergic interneurons) of subsequent afferent input, likely via PAD. An effect of SCS on presynaptic inhibition would also be consistent with its efficacy at treating spasticity (Hofstoetter *et al* 2014).

These results have several practical implications. First, it is worth emphasizing that SCS patterns optimized for driving muscles directly may not be optimal for generating subthreshold facilitation of spinal circuits. In future, computational models such as the one we have introduced may be useful for designing temporally-patterned stimulus trains to maximize synaptic facilitation while minimizing the gating effects of presynaptic inhibition. Alternatively, using the facilitation of cortical-evoked muscle responses (for example by transcranial magnetic stimulation) as a biomarker of corticospinal excitability may allow patient-specific stimulation parameters to be optimized. Finally, the effect of GABA_B_ agonists that are commonly used to treat spasticity after SCI should be considered when developing and evaluating SCS therapies, since these have indirect effects on pre-synaptic inhibition (Rudomin and Schmidt 1999).

In summary, we have shown that intermediate-frequency trains of subthreshold dorsal epidural SCS and transcutaneous SCS can raise the excitability of spinal circuits and thus enhance upper-limb muscle responses to weak supraspinal inputs. By contrast, ventral epidural SCS is effective at activating those muscles directly, even with low current intensities and high stimulation frequencies. Both effects may be useful in the restoration of upper-limb function following SCI, and the epidural cuff electrode used here may pave the way for clinical implants to deliver both types of stimulation in patients. Consideration of the interaction between synaptic facilitation of motoneurons and presynaptic gating mechanisms will help design stimulation protocols to maximize the potential of these therapies.
